# Activin A Inhibits Antigen-Induced Allergy in Murine Epicutaneous Sensitization

**DOI:** 10.3389/fimmu.2013.00246

**Published:** 2013-08-22

**Authors:** Magdalini Kypriotou, Dianelys Rivero, Sergio Haller, Anita Mariotto, Marcel Huber, Hans Acha-Orbea, Sabine Werner, Daniel Hohl

**Affiliations:** ^1^Laboratory of Cutaneous Biology, Service of Dermatology and Venereology, Beaumont Hospital, CHUV, Lausanne, Switzerland; ^2^Department of Biochemistry, University of Lausanne, Lausanne, Switzerland; ^3^Department of Biology, Institute of Molecular Health Sciences, ETH Zurich, Zurich, Switzerland

**Keywords:** activin, epicutaneous sensitization, transgenic mice, inflammation, atopy

## Abstract

Activin A, a member of the TGFβ superfamily, is involved in physiological processes such as cell differentiation, tissue homeostasis, wound healing, reproduction, and in pathological conditions, such as fibrosis, cancer, and asthma. Activin enhances mast cell maturation, as well as regulatory T-cell and Langerhans cell differentiation. In this study we investigated the potential role of activin in epicutaneous sensitization with ovalbumin (OVA), notably with respect to its effect on known Th2-polarization. For this purpose, transgenic mice overexpressing activin in keratinocytes and their wild-type (WT) controls were sensitized epicutaneously with OVA. Skin biopsies were analyzed with regard to histopathological features and mRNA expression of pro-inflammatory and Th1/Th2 cytokines, and Ig levels were measured in the serum. Unexpectedly, activin overexpressing animals were protected from Th2-cytokine expression and induction of OVA-specific IgE levels compared to WT animals. On the other hand, transgenic mice were more susceptible to inflammation compared to WT littermates after tape-stripping and saline (vehicle) or OVA application, as shown by increased pro-inflammatory cytokine mRNA levels and neutrophil accumulation at the site of the treatment. We conclude that activin protects from antigen-induced cutaneous Th2-polarization through modulation of the immune response. These findings highlight the role of activin in cutaneous sensitization, allergy, and in skin homeostasis.

## Introduction

Activins belong to the “transforming growth factor” (TGFβ) superfamily of cytokines and growth factors. The most abundant activin variant is activin A, the homodimer formed by two βA subunits connected by a disulfide bridge (1, 2). In addition to the well-characterized dimers, activin A (βAβA), B (βBβB), and AB (βAβB), two additional β chains (βC and βE) have been described. Activins exert their biological effects through activation of transmembrane serine/threonine kinase receptors. Binding to a type II activin receptor (ActRIIA or ActRIIB) leads to the recruitment, phosphorylation, and activation of a type I activin receptor (ActRIB = Alk4, or ActRIC = Alk7). This activates the canonical signaling pathway *via* Smad proteins (Smad2/3/4; *C. Elegans* SMA and Mothers against decapentaplegic homologs), or, alternatively, mitogen-activated kinase pathways (MAPK). Activin bioavailability is regulated by natural soluble inhibitors, such as follistatin, follistatin-related protein (FRP), and inhibin, and by membrane-bound proteins, such as betaglycan (in complex with inhibin), crypto, and BAMBI (3). Activins were initially described as reproductive hormones, but they also have important functions in development, tissue homeostasis, and repair. Activins enhance fibrosis and epidermal skin cancer, promote bone and skin wound repair, and act as pro- or anti-inflammatory proteins in a cell type and organ dependent manner. Thus, it is not surprising that abnormalities in activin receptor expression and/or signaling are associated with various human diseases (4, 5). Activin has immunomodulatory functions and can have pro- or anti-inflammatory activities (3, 5, 6). In skin, activin overexpression enhances carcinogenesis through generation of a pro-tumorigenic immune cell response (4). In addition, mice overexpressing activin in keratinocytes (K14-Activin tg) of the epidermis and hair follicles present abnormal keratinocyte differentiation in the tail skin, enhanced wound repair and increased populations of regulatory T-cells (Treg) (7–9). Further, activin induces Langerhans cell (LC) differentiation (10), and follistatin overexpression in the epidermis of transgenic mice leads to reduction of the LC population (11). Finally, activin induces mast cell maturation and migration (12). Thus, activin is linked to particular biological processes, known to be involved in atopy, which made us wonder whether it could exert an active role in atopic dermatitis (AD).

Atopic dermatitis is the most common inflammatory skin disease, affects 10–20% of children and 1–3% of adults, and predisposes to asthma and allergic rhinitis (13). AD origins involve genetic, immune, and environmental factors. Briefly, impaired barrier function, which in 50% of the cases is caused by profilaggrin (*FLG*) gene mutations, favors the penetration of irritants and allergens through the epidermis. This leads in the predisposed host to activation of LC, and subsequently to an initial Th2 cell polarization induced by specific cytokines, such as thymic stromal lymphopoietin (TSLP) (14). Recently, activation of intraepithelial lymphocytes (dendritic epidermal T-cells in mice, DETCs) by antigens expressed on stressed keratinocytes has been shown to contribute to Th2 cell polarization (15). Migration of inflammatory cells to the site of lesion and specific cytokine accumulation result in a Th2 to Th1 switch and the “chronic” phase of AD, characterized by keratinocyte hyperproliferation and epidermal thickening with prominent infiltrates of LC, eosinophils, monocyte-macrophages, and mast cells (16).

There is no current information about activin involvement in human skin allergic diseases. However, this cytokine is strongly associated with allergic asthma, one of the atopic diseases (17). Airway epithelial barrier presents several common functional features and immune reactivity with epidermal barrier. Therefore, we undertook an *in vivo* study which investigates the potential role of activin in a mouse model for allergic dermatitis (18, 19). Repeated epicutaneous sensitization with ovalbumin (OVA) leads to AD-like lesions in mice characterized by progressive thickening of dermis and epidermis, and the presence of inflammatory infiltrates in the skin. Further, epicutaneously OVA-treated mice develop an early Th2-polarization, characterized by increased mRNA levels of IL4, IL13, and IL5, and high levels of IgE and OVA-specific IgE, which is another common feature of acute human AD. After 3 weeks of OVA exposure, increased mRNA levels of IFNγ, IL12p35, and OVA-specific IgG2a are observed, which is reminiscent of the Th2 (acute phase) to Th1 (chronic phase) switch of human AD (18, 20).

Ovalbumin sensitization of transgenic mice overexpressing activin in keratinocytes led to Th2-independent inflammation. Our data reveal that activin plays a protective role against antigen-specific dermatitis and suggest that it is involved in the onset of AD-like symptoms in an epicutaneous sensitization mouse model.

## Materials and Methods

### Mice – OVA-epicutaneous sensitization

Wild-type (WT) CD1 (Crl:, Charles River, France), and K14-Activin transgenic mice (K14-Act mice) (2, 4) were housed, fed, and bred under SPF (Specific Pathogenic Free) conditions, according to federal guidelines and the federal and local authorities approved procedures. Breeding was performed between WT female and K14-Act tg male mice. WT and K14-Act tg female littermates were used for the experiments. Four to six-week-old female mice were treated according to the OVA-epicutaneous sensitization protocol described previously (18). Briefly, at day 0 (d0), mice were anesthetized, shaved and tape-stripped 12 times. A 1 cm^2^ piece of sterile gauze containing 100 μl of OVA (Sigma, Switzerland) (1 mg/ml in NaCl 0.9%) or 100 μl of NaCl 0.9% only (control – Ctl) was secured on the back with a bandage, left for 7 days, then removed, this process followed by a 14-day rest period. Epicutaneous sensitization with OVA was repeated three times. The mice were sacrificed on day 50 (d50), and skin biopsies and serum were collected. Four groups of mice [WT – vehicle-treated (Ctl), WT OVA-treated, K14-Act vehicle-treated (Ctl), and K14-Act OVA-treated], each including 10–15 mice, were used.

### RNA extraction and real-time RT-PCR

Total RNA from dorsal skin mouse biopsies was extracted using the RNeasy Fibrous Tissue Mini Kit (Qiagen, Germany). RNA integrity was verified on an agarose gel under denaturating conditions. RNA (2 μg) was reverse-transcribed into cDNA using MMLV-reverse transcriptase (New England Biolabs, UK) as follows: 10 min at 25°C, 60 min at 42°C, 5 min at 95°C. Real-time PCR analysis was performed on a StepOne™ PCR apparatus (Applied Biosystems, UK) using a Power SYBR Green Master Mix (Applied Biosystems, UK) and specific primers. Samples were amplified as described (21). The primers were designed using the Roche software (Universal Probe Library, Assay Design Center) unless described differently (Table 1). Analysis of relative gene expression was performed using the 2^−ΔΔCT^ method (22). Hypoxanthine guanine phosphoribosyl transferase (*Hprt*) mRNA was used as internal control. Statistical analysis was performed with the Mann–Whitney *U*-test.

**Table 1:** **Real-time PCR primers used in this study**.

Target gene	Forward	Reverse	GenBank accession number
Il1β	TGAAGTTGACGGACCCCAAA	TGATGTGCTGCTGCGAGATT	NM_008361
Tnfα	CCAGGCGGTGCCTATGTCT	GGCCATTTGGGAACTTCTCAT	NM_013693
Tslp	TCCTATCCCTGGCTGCCCTTCA	TGTGCCATTTCCTGAGTACCGTCA	NM_021367
Il4	CATCGGCATTTTGAACGAG	CGAGCTCACTCTCTGTGGTG	NM_021283
Il13	CCTCTGACCCTTAAGGAGCTTAT	CGTTGCACAGGGGAGTCT	NM_008355
Ifnγ	ATCTGGAGGAACTGGCAAAA	TTCAAGACTTCAAAGAGTCTGAGGTA	NM_008337.3
Il10	CAGAGCCACATGCTCCTAGA	GTCCAGCTGGTCCTTTGTTT	NM_010548.1
Il17A	ACCCTGGACTCTCCACCGCA	GTGCAGCCCACACCCACCAG	NM_010552.3
Foxp3	AGAAGCTGGGAGCTATGCAG	GCTACGATGCAGCAAGAGC	NM_054039.1
Rae-1	TGGACACTCACAAGACCAATG	CCCAGGTGGCACTAGGAGT	NM_020030
Hprt	GTTGGATACAGGCCAGACTTTGTTG	GATTCAACTTGCGCTCATCTTAGGC	NM_013556

### Histology

Dorsal skin biopsies were fixed overnight in freshly prepared PFA 4% (paraformaldehyde) at 4°C, then washed in TBS and embedded in paraffin. Skin specimens of 5 μm thickness were deparaffinized, rehydrated, and stained with hematoxylin and eosin as described (21). Stained sections were analyzed under the light microscope (Nikon Ellipse E400, Switzerland) coupled to a CCD camera.

### Immunofluorescence

Dorsal skin biopsies were snap frozen in isopentane, embedded in OCT and cut in 5 μm cryosections. Specimens were fixed in ice-cold acetone, blocked with 5% NGS (normal goat serum) – TBS – GBA (glycine – BSA) for 1 h and incubated at room temperature (RT) with the primary antibody – γδTCR-FITC antibody (BD Biosciences San Diego, CA), anti-MPO (myeloperoxidase) (Neomarkers, Thermo Scientific, Fremont – CA, USA), anti-F4/80 (Caltag Invitrogen AG, Camarillo, CA, USA), or anti-langerin (CD207, clone 929F3.01, Dendritics, France). Three hours later, slides were rinsed and incubated for 1 h with the appropriate secondary antibody (Alexa Fluor 488 IgG, Molecular Probes, Invitrogen, Netherlands), when needed, then counterstained with DAPI and mounted with a fluorescent mounting medium (Dako, Denmark). The images were captured by a confocal microscope (LSM 700 Zeiss, Switzerland) and analyzed using the ZEN2010 software.

γδTCR-, MPO-, F4/80-, or langerin-positive cells were counted in 10–15 microscope fields (×20) per animal (five mice per group), and expressed as cells per millimeter of basal layer or per square millimeter of skin. Numbers in vehicle-treated WT animals were used as reference. Mean and SD were calculated and statistically significant differences were calculated with Mann–Whitney *U*-test.

### ELISA

Detection of total IgE in serum was performed using a mouse IgE ELISA Set (BD OptEIA, BD Biosciences Pharmingen, Belgium). For OVA-specific IgE, a 96-well ELISA plate was coated with OVA (40 μg/ml) in sodium carbonate coating buffer overnight at 4°C. After blocking with 10% heat inactivated FBS, mouse serum samples (diluted 1:20 for IgE) were added and incubated for 3 h at RT. Biotinylated detection antibodies (rat anti-mouse IgE – clone R35-118, BD Pharmingen, Belgium) and a streptavidin-HRP reagent were used for detection.

Total serum Ig2a levels were detected using a sandwich ELISA: briefly, the plate was coated with a capture antibody (rat anti-mouse IgG2a – clone R11-89, BD Pharmingen) overnight at 4°C, and serum samples were diluted 1:400 and incubated for 2 h at RT. A biotinylated antibody (Rat anti-mouse IgG2a – clone R19-15, BD Pharmingen) and streptavidin-HRP reagent were used for detection.

Colorimetric reactions occurred with TMB substrate solution (Invitrogen, Netherlands) after 30 min incubation in the dark. Absorbance was measured at 450 nm corrected for absorbance at 620 nm. Statistical analysis was performed with the Mann–Whitney *U*-test.

## Results

### OVA-epicutaneous sensitization of K14-Activin tg mice leads to inflammation and epidermal thickening

**Figure 1:**
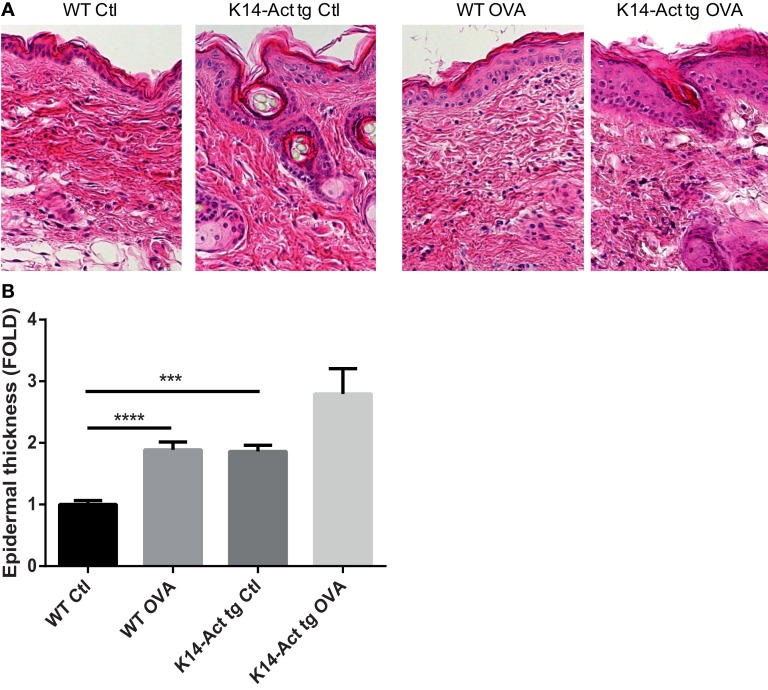
**OVA-epicutaneous sensitization leads to epidermal thickening and inflammation**. **(A)** WT and K14-Act mice were treated with vehicle (Ctl) or OVA. Skin biopsies were stained for H&E (20× objective). **(B)** Epidermal thickness (7–10 animals per group, five to seven fields per animal) in micrometer was measured on H&E-stained biopsies from the four experimental groups using a scaled ocular lens on a light microscope. Mean values are represented as fold of the mean value of WT vehicle group (Ctl) ± SEM. Statistical differences were calculated with the Mann–Whitney *U*-test (*p* < 0.05*, *p* < 0.001***).

**Figure 2:**
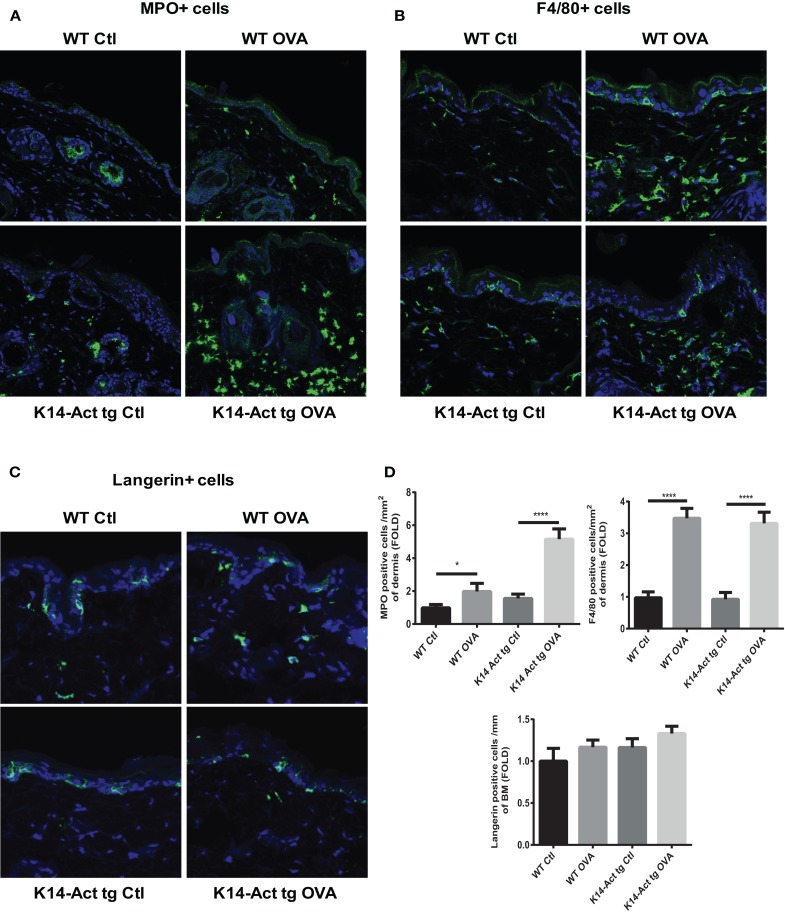
**Morphological and cell analysis of vehicle-treated and OVA-treated mice**. Cryosections from the four experimental groups were obtained. MPO+ **(A)**, F4/80+ **(B)**, and langherin+ cells **(C)** were labeled with the respective antibodies, and analyzed with a confocal microscope (20× objective). **(D)** MPO, F4/80, or langerin-positive cells were quantified and statistic analysis was performed with the Mann–Whitney *U*-test (*p* < 0.05* and *p* < 0.001***).

After treatment of WT and K14-Act mice with either OVA or vehicle, histological analysis showed important differences between the four groups of mice, in terms of epidermis thickening and immune cell populations (Figures 1 and 2). In WT OVA-treated mice, the epidermis was thickened (2-fold) compared to WT vehicle-treated mice, and displayed lymphocytic exocytosis and parakeratotic hyperkeratosis (Figures 1A,B). Further, the dermis from WT OVA-treated mice showed features of fibrosis, as well as moderate infiltrates of lymphocytes, macrophages, and to a lesser extent neutrophils (Figures 2A,B,D). Untreated back skin from K14-Act mice is phenotypically normal (4), whereas vehicle-treated skin from K14-Act mice was characterized by thickened epidermis (2-fold) and dermis compared to vehicle-treated WT mice (Figures 1A,B). Further, the epidermis of K14-Act OVA-treated mice was thickened compared to vehicle-treated WT mice (approximately 3-fold), and lymphocytic exocytosis and hyperparakeratosis were observed (Figures 1A,B). Inflammatory infiltrates, essentially composed of neutrophils, were more abundant in the dermis of K14-Act OVA-treated mice compared to the rest of the groups (Figure 1A; Figures 2A,D). Numbers of LC and *Foxp3* mRNA, as an indicator of the Foxp3+ Treg cell population in skin, were not altered at the end of the protocol in any group (Figures 2C,D, and data not shown).

**Figure 3:**
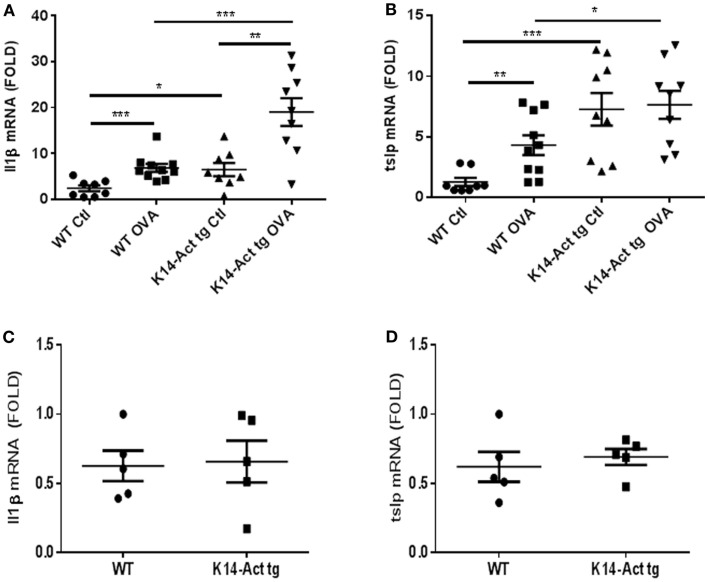
**Expression of pro-inflammatory cytokines in vehicle and OVA-treated mice**. cDNAs were generated from WT and K14-Act mice and real-time PCR was performed. *mIL1*β **(A,C)** and *mTslp* **(B,D)** mRNAs were amplified using cDNAs from untreated WT and K14-Act mice **(C,D)** or treated with vehicle or OVA **(A,B)**. Ct values were normalized to expression levels of the *hprt* house-keeping gene. The experiment was realized in groups of five mice for the untreated and 8–10 mice for the vehicle- or OVA-treated mice. Results are presented in a scatter graph and mean values ± SEM are shown. Statistical differences were calculated with the Mann–Whitney *U*-test (*p* < 0.05*, *p* < 0.01**, and *p* < 0.001***).

The expression of IL1β and TNFα was analyzed in order to further characterize the local inflammatory response. In addition, we analyzed the expression of TSLP, a marker cytokine for impaired barrier function and inducer of Th2-polarization. No differences in *Il1*β, *Tnf* α, or *Tslp* mRNA levels were observed between untreated WT and K14-Act mice (Figures 3C,D, and data not shown). However, *Il1*β and *Tslp* mRNAs were significantly increased in vehicle-treated K14-Act mice as compared to vehicle-treated WT mice (Figures 3A,B). No changes on *Tnfa* mRNA were observed between these two groups (data not shown). Obviously, the enhanced expression of pro-inflammatory cytokines results from the experimental protocol, including shaving, tape-stripping, and bandage application, which induces a non-specific inflammatory response to mechanical barrier injury and vehicle application. *Il1*β and *Tslp* mRNAs were increased in the OVA-treated WT mice compared to the vehicle-treated WT group (Figures 3A,B). *Tnfa* mRNA was also mildly increased in the OVA-treated WT mice compared to the saline-treated WT animals, but there was no other significant difference when compared to other groups (data not shown). Inflammation in vehicle- and OVA-treated transgenic mice was associated with high local mRNA expression levels of both *Il1*β and *Tslp* mRNAs, when compared to vehicle-treated WT group (Figures 3A,B). However, while antigen application did provoke a strong *Il1*β mRNA increase in K14-Act mice compared to the saline-treated K14-Act, *Tslp* mRNA was already elevated without OVA, and no further increase was noted by allergen sensitization (Figures 3A,B). These results reveal that repeated vehicle and OVA application provoked a differential cutaneous reaction, which was translated into a divergent cytokine expression pattern. Further, our data imply that activin overexpressing animals were more susceptible to inflammation after mechanical stress.

### Reduced Th2-polarization in K14-Activin mice

**Figure 4:**
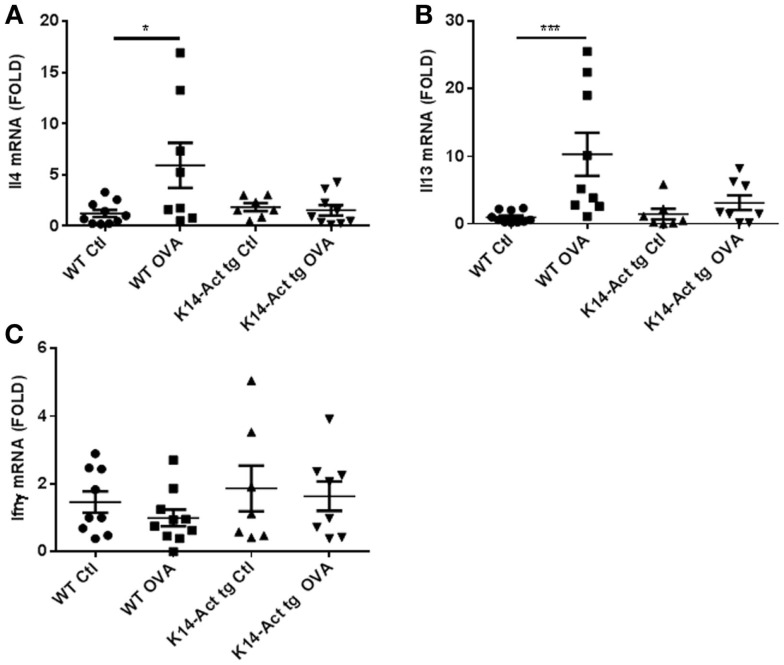
**Th2-specific cytokines increase upon OVA treatment of WT mice but not of K14-Act mice**. Skin biopsies from WT and K14-Act mice, untreated or treated (vehicle-Ctl or OVA) were collected and used for RNA isolation and subsequent generation of cDNA. Real-time PCRs were performed using primers specific for **(A)** *mIl13*, **(B)** *mIl4*, and **(C)** *mIfn*γ. Ct values were normalized to expression levels of the *hprt* house-keeping gene. The experiment was realized in groups of 8–10 mice. Results are presented in a scatter graph and mean values ± SEM are shown. Statistically significant differences were calculated with the Mann–Whitney *U*-test (*p* < 0.05*, *p* < 0.01**, and *p* < 0.001***).

Acute AD involves a hypersensitivity response associated with Th2-polarization. Surprisingly, *Il13* and *Il4* mRNAs remained unchanged in the K14-Act OVA-treated mice compared to vehicle-treated WT and K14-Act mice. This is an activin-dependent effect because the mRNAs of the same cytokines were increased in WT OVA-treated mouse skin, as expected. These results suggest that activin prevents a Th2-response to OVA stimulation (Figures 4A,B).

*Ifn*γ mRNA expression, as a marker of Th1 response in skin appearing in chronic AD stages, was similar for all groups of animals (Figure 4C) Consequently, these mice did not show signs of chronic eczematous lesions during the experimental protocol.

### Activin overexpression hampers the production of IgEs

**Figure 5:**
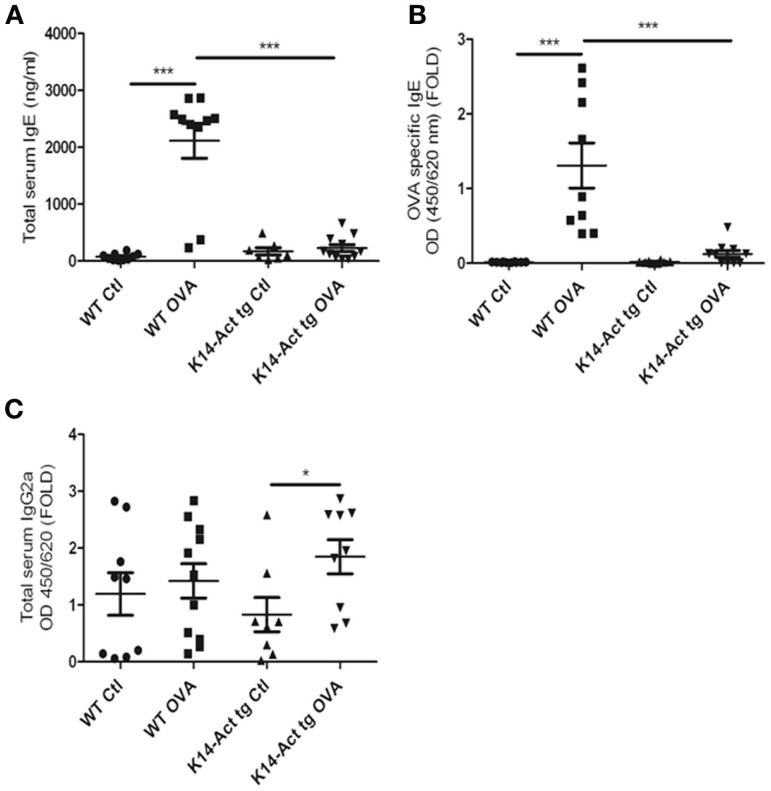
**Activin overexpression inhibits the production of IgEs after OVA treatment**. WT and K14-Act mice treated with OVA or vehicle were sacrificed, and serum was collected. Total IgE **(A)**, OVA-specific IgE **(B)**, and total IgG2a **(C)** ELISA were performed as described in Section “Materials and Methods.” The data are presented as a scatter plot and mean values are shown (*n* = 8–12). Statistically significant differences were calculated with the Mann–Whitney *U*-test (*p* < 0.05* and *p* < 0.01**).

To better dissect the involvement of activin in the OVA-epicutaneous sensitization, serum IgEs and IgG2a levels were measured. Serum IgEs were significantly increased only in the WT OVA-treated mice, whereas they remained at the basal level for the other three groups (Figure 5A). The discrete and non-significant increase in the vehicle-treated and OVA-treated K14-Act mice in comparison to the vehicle-treated WT animals indicates a role of mechanical barrier disruption in atopic sensitization.

Ovalbumin-specific IgEs were increased only in the WT-OVA mice group, and barely detectable in OVA-treated transgenic mice (Figure 5B). Thus, activin inhibits the antigen-specific immune response in this experimental setting.

Further, a minor increase was observed in total serum IgG2a, for the OVA-treated K14-Act group compared to the K14-Act Ctl mice, although OVA-specific IgG2a were not detectable (Figure 5C and data not shown). Thus, activin is involved in non-specific polyclonal IgG2a modulation, without inducing a Th1-polarization, which should be associated with higher levels of antigen-specific IgG2a and increased concentrations of Ifnγ. In sum, activin overexpression hampers the immune response by blocking IgE production.

### Depletion of γδ T-cells from K14-Act mouse epidermis after OVA sensitization

**Figure 6:**
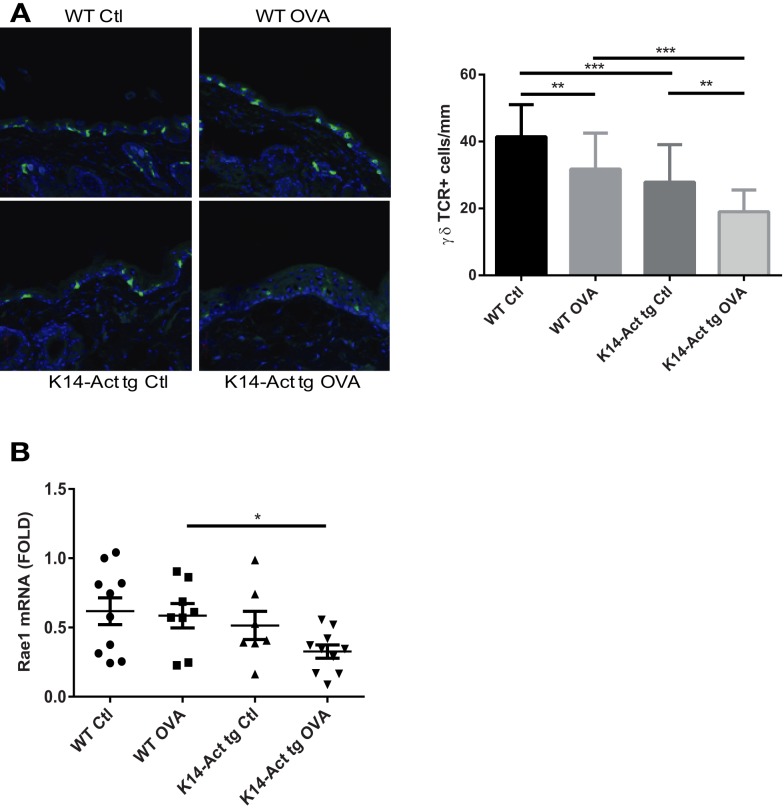
**Activin overexpression causes γδ T-cell depletion in the epidermis after mechanical injury and allergic sensitization**. **(A)** Dorsal skin biopsies from the four experimental groups were collected, and cryosections were obtained. Epidermal γδ-T-cells were analyzed with a confocal microscope (20× objective). Stained cells were quantified and statistical analysis was performed with the Mann–Whitney *U*-test (*p* < 0.01 for ** and *p* < 0.001 for ***). **(B)** Real-time PCR was performed using primers specific for *mRae-1*. Ct values were normalized to expression levels of the *hprt* house-keeping gene. The experiment was realized in groups of 8–10 mice for the sensitized mice. Results are presented in a scatter graph and mean values ± SEM are shown. Statistically significant differences were calculated with the Mann–Whitney *U*-test (*p* < 0.05*).

γδ T-cells are involved in the induction of Th2-response and IgE production after tissue damage (23). To test whether activin may involve γδ T-cell mediated immune surveillance in this experimental setting, γδ T-cells were quantified. A strong reduction of the epidermal γδ T-cell population was noted in K14-Act animals, particularly after OVA treatment (Figure 6A). Further, mRNA levels of *Rae1* (Retinoic acid early 1), an NKG2D ligand, were found significantly decreased in the K14-Act OVA group when compared to the WT OVA mice (Figure 6B). Given that epidermal γδ T-cell numbers in untreated K14-Act mice as compared with WT mice are unaffected (4), our results suggest that activin obstructs maintenance of γδ T-cells in hyperproliferative epidermis after mechanical injury, and this is further enhanced by antigen exposure. This significant depletion of the γδ T-cell population in the K14-Act mice after epicutaneous sensitization may contribute to the reduced allergic reaction.

### Upregulation of *Il10* and *Il17* mRNA expression in OVA-treated transgenic mice

**Figure 7:**
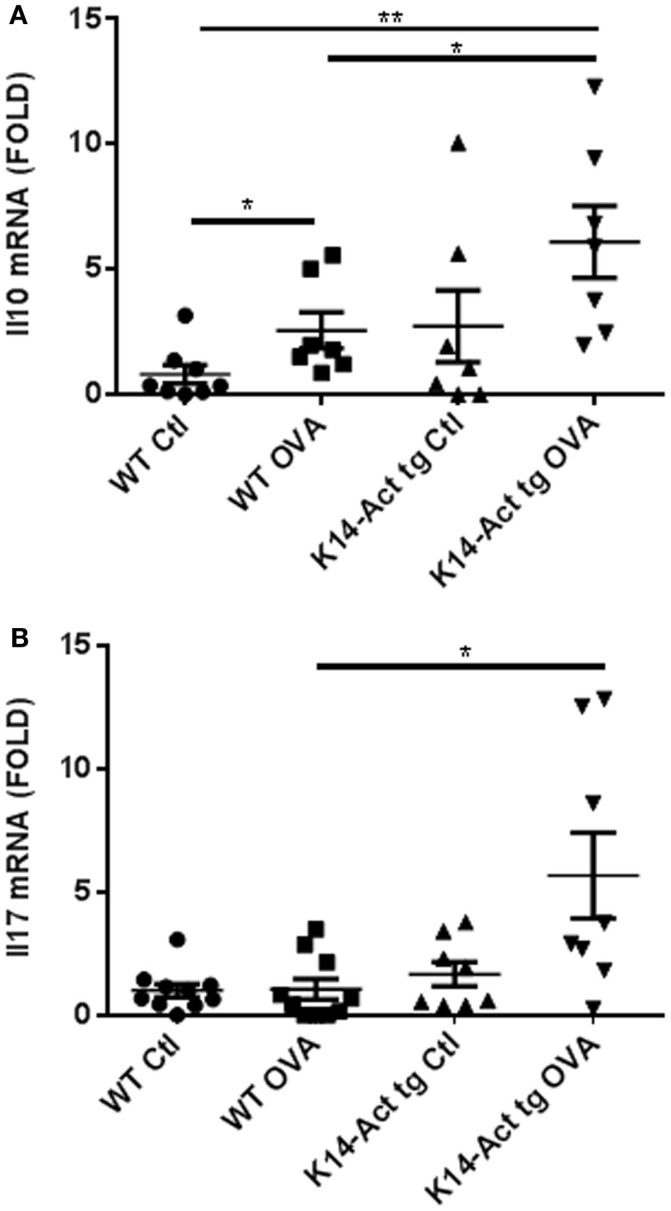
***Il10* and *Il17A* mRNA expression is increases in OVA-treated K14-Act mice**. Skin biopsies from WT and K14-Act mice, vehicle, or OVA-treated, were collected and used for RNA isolation and subsequent generation of cDNA. Real-time PCR was performed using primers specific for *mIl10* **(A)** and *mIl17A* **(B)**. Ct values were normalized to expression levels of the *hprt* house-keeping gene. The experiment was realized in groups of 7–10 mice. Results are presented in a scatter graph and mean values ± SEM are shown. Statistically significant differences were calculated with the Mann–Whitney *U*-test (*p* < 0.05*, *p* < 0.01**).

IL10’s main role is to suppress allergic responses, but it can also be a Th2-effector cytokine, according to the molecular context (24, 25). Its expression was evaluated in dorsal skin biopsies obtained from vehicle or OVA-treated mice, by real-time PCR. Interestingly, *Il10* mRNA expression was increased in OVA-treated K14-Act mice compared to OVA-treated WT mice (Figure 7A). IL10 secretion in the absence of Th2 cytokines, as for the OVA K14-Act mice (Figure 4), strongly suggests a regulatory function, which could explain the suppression of Th2-response and IgE production. IL10 mRNA expression is also increased in OVA-treated WT mice when compared to Ctl WT mice; in this case, IL10 could be co-secreted from Th2-cells (Figure 7A).

IL17A, mainly produced by Th17 cells but also by neutrophils and other immune cells, was measured as a marker of tissue inflammation and eventual TSLP-induced Th2-polarization inhibitor (26). *Il17A* mRNA was significantly increased in OVA-treated K14-Act skin biopsies compared to OVA-treated WT samples, suggesting the involvement of Th17-related cytokines in allergen epicutaneous sensitization (Figure 7B).

## Discussion

In this study we demonstrated for the first time that epidermal keratinocyte-derived activin protects from antigen-specific T-cell immune response in an epicutaneous sensitization murine model, because: (1) It inhibited Th2-polarization after antigen-induced allergic dermatitis, (2) It hampered IgE and antigen-specific IgE production in mouse serum, and (3) It led to decrease of γδ T-cell population in injured skin. These findings are novel and they provide further evidence about the role of activin in skin, already known for its involvement in wound healing and, skin morphogenesis and tumorigenesis (2, 4, 9, 27).

Ovalbumin-epicutaneous sensitization induces a Th2-specific response characterized by high IgE production (19). This response evolves during the time of treatment, and depends on the quantity of the antigen absorbed (19, 20, 28). Activin strongly blocked this response because in OVA sensitized K14-Act transgenic animals, Th2-cytokine expression levels remained low, and total IgE and antigen-specific IgE serum levels were not significantly changed compared to vehicle-treated mice. In contrast, antigen-treated WT mice developed high total and OVA-specific IgE responses, associated with an increase in Th2-derived *Il4* and *Il13* mRNA expression, as expected.

The mechanism through which activin regulates antigen sensitization responses in skin is not elucidated. However, it is known that activin is involved in the antigen presentation process (4, 8, 25, 29, 30). Further, activin A suppresses effector Th2, but also Th1 immunity through generation of a specific subset of regulatory T-cells (25). Treg subsets regulate T-lymphocyte activation and suppress eosinophil and mast cell responses, inducing allergen peripheral tolerance and protecting from anaphylactic shock (31). Activin-induced Tregs in mouse lung draining lymph node (DLN) are CD4 + CD25-Foxp3−, express IL10 and TGFβ1 and inhibit CD11c+ DCs maturation (7, 8, 25). Indeed, in our experimental setting, *Il10* mRNA was increased in the OVA-treated K14-Act mice, which, in the absence of Th2-responses, may suggest the action of Treg cells. Except from Treg cells, IL10 is also secreted by macrophages, neutrophils, fibroblasts, and keratinocytes, together with other cytokines, such as TNFα, IL1β, IL8, and IL6, as a consequence of DNA damage or inflammation (32–34). However, the specific cytokine and cell type profile detected in this study strongly supports the immunosuppressive role of IL10 in these experimental conditions. The above data suggest that activin inhibits Th2-polarization in our model, through a Treg-dependent mechanism. The absence of a local Th1-polarization, in our study, does not exclude a broader activin-mediated suppressive effect on T-cell responses.

This is the first report providing data about the function of activin in antigen-induced cutaneous allergic reaction, which are consistent with its protective role from atopic pulmonary disease (17, 25). Activin is increased in serum and in CD4+ T-cells of patients with mild asthma, although there are no differences between healthy subjects and severely affected patients (25, 35). It is mainly secreted by immune, lung smooth muscle and epithelial cells and enhances epithelial and alveolar cell proliferation, promoting tissue remodeling after allergen exposure (35–38). In parallel, it is involved in the maturation of DCs and diminishes their antigen presenting capacity to T-cells (30), indicating that activin suppresses allergic reactions and maintains tissue homeostasis.

Barrier disruption-induced Th2-polarization is often preceded by increase of TSLP expression (28, 39). TSLP is an epithelial-derived cytokine and signals through a heteromeric receptor (TSLPR and IL7rα). It is involved in the activation and/or proliferation of several immune cell populations, such as B and T lymphocytes, basophils and eosinophils, and is essential for DC-driven Th2-polarization after mechanical injury of the epidermal barrier (28, 40, 41). *TSLP* gene transcription is activated by IL1β and TNFα (42). In our experimental setting, *Tslp* mRNA expression was increased in all three experimental groups, compared to WT Ctl mice, independently on the presence or not of antigen. TSLP upregulation is likely to be independent on the IL1β increase during epicutaneous sensitization, because the expression levels of their mRNAs did not correlate in the different experimental groups (Figures 3A,B). Indeed, Oyoshi et al. (28), showed a rapid and transient accumulation of TSLP in skin after tape-stripping. This suggests that different mechanisms are at work in our model in order to maintain TSLP expression in skin in a chronic manner.

These observations lead to the conclusion that activin plays a role downstream of TSLP and prevents Th2-cytokine expression. Bogiatzi et al. (26) recently showed that TSLP’s capacity to induce a Th2-response depends on the cytokine milieu, and is inhibited when Th17-related cytokines are present. In line with this finding, IL17, IL1β, and Smad signaling are positively modulated in OVA-treated K14-Act tg mice in our experimental setting, suggesting that activin intervenes during early immune responses.

Significant reduction of γδ T-cell population in vehicle and OVA-treated K14-Act skin, compared to WT Ctl mice, is another feature of the activin-induced Th2 allergic reaction inhibition. Indeed, γδ T-cells were shown to be required for the upregulation of Th2-derived cytokines after epicutaneous antigen sensitization, and they dramatically modulate the production of total and antigen-specific IgE (23). This link between lymphoid stress surveillance and atopic response, is further associated to Rae-1, a NKG2D membrane receptor ligand, which promotes γδ T-cell activation (23). γδ T-cells or DETCs reside in the murine epidermis and are activated after epithelial stress. They induce the elimination of damaged keratinocytes and are involved in the activation of adaptive immunity (43, 44). Intraepidermal γδ T-cells in human skin contribute to efficient wound repair, although they are more scarce compared to mouse skin (45). The decrease of intraepidermal γδ T-cells in vehicle-treated K14-Act mice, and most importantly in the OVA-treated group is consistent with reduced expression levels of *Rae-1* mRNA. Thus, activin may not only affect γδ T-cells, but also the expression of the activating ligand on keratinocytes. Conversely, the γδ T-cell population (and *Rae-1* expression) in WT mice, was not affected. Although it was shown that activin impairs human NKT cell functions without significantly modulating NKG2D receptor expression, little is known about the effect of activin on γδ T-cell receptors (29). We showed that γδ T-cells express activin receptors, but we could not detect increased apoptosis of these cells in the presence of high levels of activin during DMBA-TPA tumorigenesis. Rather, proliferation of γδ T-cells was inhibited under these conditions (4). We propose that activin also attenuates γδ T-cell function after tape-stripping, probably by inhibiting their proliferation, thereby leading to their depletion.

In this study, we also demonstrate the pro-inflammatory role of activin after epithelial barrier injury. Indeed, *Il1*β mRNA levels were increased in OVA-treated mice, and correlated with macrophage accumulation at the site of the treatment. This was consistent with previous reports demonstrating that *Il1*β and *Tnfa* are the first cytokines induced after mechanical injury and epicutaneous sensitization (20, 28, 46). In fact, activin overexpression triggers an inflammatory response in the mice treated only with saline, suggesting that they are more susceptible to mechanical disruption of the epidermal barrier. Indeed, activin A is a component of the innate immune response and triggers pro-inflammatory cytokine secretion in experimental inflammation (47). Immune responses involve very diverse and complex molecular mechanisms and cell types, and depend on the nature of “aggression,” in order to protect the organism. Activin plays a central role in defense and is controlled by a well-defined cell and cytokine milieu (5). Accordingly, activin A’s pro-inflammatory effect is dose and anatomical site-dependent (25, 48) and linked to the genetic background. CD1 K14-Act mice present spontaneous eye inflammation and periocular skin lesions at 5–6 months after birth. When backcrossed into an inbred background, such as BALB/c, the inflammatory lesions emerged earlier and also appeared at other body sites, and these manifestations were accelerated with generations. Skin biopsies from the affected regions of BALB/c K14-Act mice showed macrophage and neutrophil accumulation, but absence of eosinophils (MK and DH unpublished observations).

In sum, we showed that activin protects from antigen-induced dermatitis in skin through modulation of immune responses after epicutaneous sensitization. Our results suggest that activin intervenes in at least two ways: (a) To favor secretion of pro-inflammatory cytokines after mechanical barrier disruption, and (b) to inhibit Th2-polarization and IgE generation. Further experiments in our model, including short OVA-epicutaneous sensitization followed by restimulation of DLN cells with OVA *ex vivo*, may allow to further dissect the mechanism through which activin prevents antigen-induced immune responses in skin, and to focus on early time points.

Activin bioavailability and activity are tightly controlled by a complex network of secreted and intracellular molecules. The signals initiated by activin reach gene targets through a sophisticated pathway also firmly regulated at different levels, from the membrane to the nuclear level. Exhaustive analyses dissecting this molecular network in skin are necessary in order to consider activin or downstream mediators as successful candidates in the field of treatment for atopic diseases, ruling out activin’s pro-fibrotic, pro-inflammatory, and in particular its pro-tumorigenic effects.

## Conflict of Interest Statement

The authors declare that the research was conducted in the absence of any commercial or financial relationships that could be construed as a potential conflict of interest.
